# Ethanol and Reactive Species Increase Basal Sequence Heterogeneity of Hepatitis C Virus and Produce Variants with Reduced Susceptibility to Antivirals

**DOI:** 10.1371/journal.pone.0027436

**Published:** 2011-11-08

**Authors:** Scott Seronello, Jessica Montanez, Kristen Presleigh, Miriam Barlow, Seung Bum Park, Jinah Choi

**Affiliations:** School of Natural Sciences, University of California Merced, Merced, California, United States; Institut National de la Santé et de la Recherche Médicale, France

## Abstract

Hepatitis C virus (HCV) exhibits a high level of genetic variability, and variants with reduced susceptibility to antivirals can occur even before treatment begins. In addition, alcohol decreases efficacy of antiviral therapy and increases sequence heterogeneity of HCV RNA but how ethanol affects HCV sequence is unknown. Ethanol metabolism and HCV infection increase the level of reactive species that can alter cell metabolism, modify signaling, and potentially act as mutagen to the viral RNA. Therefore, we investigated whether ethanol and reactive species affected the basal sequence variability of HCV RNA in hepatocytes. Human hepatoma cells supporting a continuous replication of genotype 1b HCV RNA (Con1, AJ242652) were exposed to ethanol, acetaldehyde, hydrogen peroxide, or *L-*buthionine-*S,R*-sulfoximine (BSO) that decreases intracellular glutathione as seen in patients. Then, NS5A region was sequenced and compared with genotype 1b HCV sequences in the database. Ethanol and BSO elevated nucleotide and amino acid substitution rates of HCV RNA by 4–18 folds within 48 hrs which were accompanied by oxidative RNA damage. Iron chelator and glutathione ester decreased both RNA damage and mutation rates. Furthermore, infectious HCV and HCV core gene were sufficient to induce oxidative RNA damage even in the absence of ethanol or BSO. Interestingly, the dn/ds ratio and percentage of sites undergoing positive selection increased with ethanol and BSO, resulting in an increased detection of NS5A variants with reduced susceptibility to interferon alpha, cyclosporine, and ribavirin and others implicated in immune tolerance and modulation of viral replication. Therefore, alcohol is likely to synergize with virus-induced oxidative/nitrosative stress to modulate the basal mutation rate of HCV. Positive selection induced by alcohol and reactive species may contribute to antiviral resistance.

## Introduction

Hepatitis C virus (HCV) is a major etiologic agent of severe liver diseases including cirrhosis and hepatocellular carcinoma that has infected about 170 million people worldwide. Current anti-HCV therapy, consisting of pegylated interferon alpha and a nucleoside analog, ribavirin, results in sustained virological response in only 50-60% of patients undergoing treatment [1], [2]. Both the efficacy of antiviral therapy and success of re-treating the resistant population are strongly affected by HCV sequence. Specific HCV factors have also been identified that can affect the sensitivity of the virus to antivirals, such as NS5A [1], [2]. In addition, HCV, like other RNA viruses, exhibits a high level of genetic variability that complicates antiviral therapy and development of vaccines. HCV variants with reduced susceptibility to antivirals can occur naturally, even before treatment begins [3]. Such pronounced basal sequence heterogeneity and generation of escape mutants remain an ongoing concern even as new antivirals and vaccine candidates are being tested [3], [4], [5]. Furthermore, alcohol decreases the efficacy of anti-HCV therapy, and alcohol use is correlated with increased sequence heterogeneity of HCV RNA in patients [6], [7]. However, how ethanol affects HCV sequences is unclear.

Ethanol metabolism generates acetaldehyde and reactive oxygen species (ROS), reactive nitrogen species (RNS), and lipid peroxidation products while decreasing glutathione (GSH) and selenium [8]. In addition, HCV induces oxidative/nitrosative stress through several mechanisms which is exacerbated by ethanol, and GSH is significantly depleted in hepatitis C patients [8], [9]. Acetaldehyde, ROS, RNS, and lipid peroxidation products can attack the bases and deoxyribose backbone of the DNA and/or RNA and may thus act as mutagen to the viral RNA [10], [11]. In addition, evolution is affected by the environment, and ethanol, reactive species, and lipid peroxidation products can also alter the intracellular milieu for viral replication by modification of cell metabolism and signaling which may cause the virus to adapt [12], [13].

The goal of this study, therefore, was to test the effects of ethanol and reactive species on the mutability of HCV RNA in hepatocytes. Genotype 1 HCV shows higher resistance to therapy. Thus, Con1 replicon of genotype 1b, which is the best characterized in terms of adaptive mutations and replicative functions, was exposed to physiologically attained and non-cytotoxic concentrations of ethanol and acetaldehyde and then analyzed for mutation trends. *L-*Buthionine-*S,R*-sulfoximine (BSO) decreases intracellular GSH by 60–75% in these cells and was also utilized in the study to test the effects of GSH depletion seen in hepatitis C patients [14], [15]. Exogenous H_2_O_2_, on the other hand, was used to observe the effects of sublethal, exogenous oxidative stress [16]. We found that ethanol and reactive species increase oxidative RNA damage and sequence heterogeneity of the HCV RNA with evidence of positive selection. RNA damage, sequence heterogeneity of HCV RNA, and positive selection could be decreased by ROS/RNS-decreasing agents. Mutation trends and possible biological consequences of these mutations are discussed.

## Results

### Ethanol and reactive species increase the mutation rate of HCV

To determine whether ethanol can affect the sequence variability of HCV RNA, Huh7 cells that support continuous replication of genotype 1b Con1 replicon RNA were incubated with ethanol (0.5% v/v), acetaldehyde (10 µM), bolus H_2_O_2_ (100 µM), or BSO (20 µM) once daily for 48 hrs [16]. Then, the RNAs were collected, and the NS5A region was cloned by reverse transcription-polymerase chain reaction, sequenced, and compared against Con1(S1179I) sequence. We focused on NS5A because of its moderate level of diversity and its role in viral replication and antiviral resistance [1], [2], [17], [18]. The T7 HCV RNA transcript, initially used to generate the replicon cells, was also sequenced to assess the basal level of RNA heterogeneity potentially rising from any heterogeneity of HCV DNA used in the *in vitro* transcription as well as errors generated during transcription and/or cloning. After subtracting the T7 baseline values, the nt. and amino acid substitution rates of control cells were determined to be 2.89×10^−4^ nt. changes per site and 2.80×10^−4^ amino acid changes per codon (Table 1). The ethanol group showed a 6 fold increase in the nt. substitution rate and an 18 fold increase in the amino acid substitution rate over the control (P<0.001). The BSO group also displayed a significant increase in the sequence variability of HCV RNA, showing approximately 4 and 12 fold increases in nt. and amino acid substitution rates, respectively (P<0.05). H_2_O_2_ and acetaldehyde did not increase the nt. substitution rates significantly (5.28×10^−4^ and 4.25×10^−4^ nt. change per site, respectively, P>0.05) but the amino acid substitution rate increased by about 10 fold with H_2_O_2_. Therefore, ethanol and conditions that mimic GSH depletion seen *in vivo* could increase the nt. and amino acid substitution rates of HCV RNA over 48 hr.

**Table 1:** Nucleotide and amino acid substitution rates in control and treatment groups a.

Treatment Group	n b	Nt. change per site^c^	Fold increase	Amino acid changeper site^d^	Fold increase	Ts/Tv	dn/ds
Control	21	2.89×10^−4^	-	2.80×10^−4^	-	3.3	0.31
Ethanol	21	1.71×10^−3^ (P<0.001)^e^	**5.9**(absolute increase of 1.42×10^−3^)	4.97×10^−3^(P<0.001)^e^	**17.8**(absolute increase of 4.69×10^−3^)	6.8	1.05
BSO	28	1.20×10^−3^ (P<0.001)^e^	**4.2**(absolute increase of 0.91×10^−3^)	3.24×10^−3^(P<0.001)^e^	**11.6**(absolute increase of 2.96×10^−3^)	3.3	0.72

a Nucleotide and amino acid sequences used in the study were compared against Con1 sequence.

b Number of sequences analyzed in each group.

c Nt. substitution rate is reported as nt. change per site, which was determined as the number of nt. substitutions divided by total number of nucleotides sequenced in each group. Data were normalized by the T7 baseline value which was 1.27×10^−3^ nt. change per site.

d Amino acid substitution rate is reported as amino acid change per site, which was determined by computing the number of amino acid substitutions divided by total number of amino acids analyzed in each group. Data were normalized by the T7 baseline which was 1.96×10^−3^ amino acid change per site. Note the total number of amino acids analyzed is 1/3 of total number of nucleotides sequenced in each group in ^c^.

e Statistically significant difference from control.

Analysis of 1,212 genotype 1b sequences in the HCV sequence database revealed that transitions account for over 83.5% of all nt. substitutions (Table S1) with the Ts/Tv ratio of 5.05. Likewise, transitions comprised the majority of the nucleotide substitutions in our control and treatment groups (Table 1 and Table S1). Ethanol increased transitions more than transversions such that the T_s_/T_v_ ratio rose from 3.3 to 6.8.

### Ethanol, reactive species, and oxidative RNA damage

**Figure 1:**
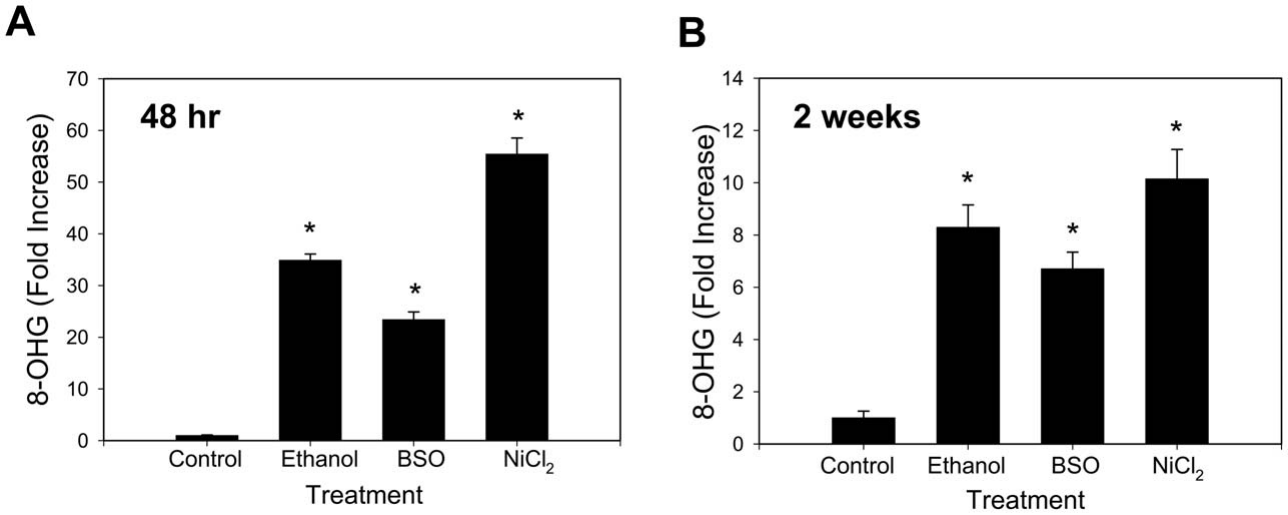
Ethanol, BSO, and oxidative RNA damage. (**A**) Con1 replicon cells were incubated with 0.5% (v/v) ethanol, 20 µM BSO, or 250 µM NiCl_2_ daily for 48 hr and analyzed for 8-OHG by ELISA. (**B**) Con1 replicon cells were incubated with 0.1% (v/v) ethanol, 20 µM BSO, or 50 µM NiCl_2_ daily for 2 weeks and analyzed for 8-OHG by ELISA. Data were expressed as fold increase from the controls (0.134±0.013 ng/ml for panel A and 0.418±0.109 ng/ml for panel B). * Indicates statistically significant difference from controls (n = 4 in panel A, n = 6 in panel B).

The observation that BSO had a greater effect on the HCV sequence than exogenous H_2_O_2_ or acetaldehyde suggested that there was a substantial level of endogenous oxidative/nitrosative stress in these cells and that endogenous ROS/RNS may have a greater effect on the HCV RNA than acetaldehyde. Therefore, to substantiate the presence of oxidative/nitrosative stress and to test the potential role of oxidative RNA damage in the accelerated mutation rate of HCV in ethanol and BSO groups, we measured 8-OHG; 8-OHG is the best-characterized and abundant form of ROS/RNS-generated RNA species [10], [11]. NiCl_2_, a positive control mutagen [19], induced a 55.4±3.1 fold increase in 8-OHG content over the control (P<0.05) (Fig. 1A). In addition, ethanol and BSO increased the level of 8-OHG by 34.9±1.2 and 23.4±1.5 folds, respectively (P<0.05) (Fig. 1A). Therefore, ethanol and reactive species could damage the RNA under the same conditions that elevated the sequence variability of HCV RNA. Daily treatment of the replicon cells with 0.1% ethanol (v/v) or 20 µM BSO for 2 weeks likewise led to significant increases in 8-OHG (Fig. 1B). The 0.1% ethanol is equivalent to blood alcohol concentration of 17.2 mM, which is approximately the legal limit for driving under the influence in many countries.

**Figure 2:**
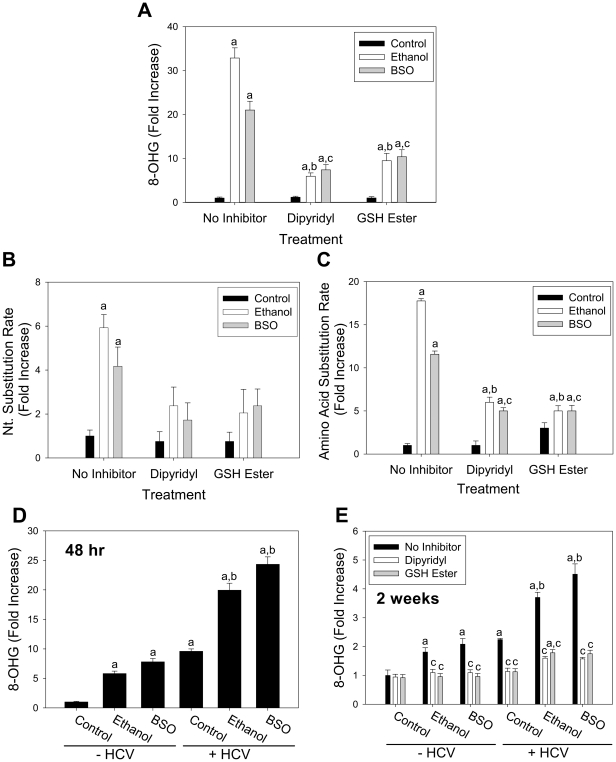
Effects of dipyridyl and GSH ester on the oxidative RNA damage and HCV mutation rate. (**A-C**) Con1 replicon cells were incubated with 0.5% ethanol or 20 µM BSO daily for 48 hr±10 µM dipyridyl or 2 mM GSH ester and analyzed for 8-OHG by ELISA (A) or sequenced and analyzed for nucleotide (B) and amino acid substitution rates (C). Data were expressed as fold increase from the control where the control values were 0.122±0.027 ng/ml (n = 6) for panel A, 2.88±0.77×10^-4^ nt. changes per site for panel B, and 2.80±0.61×10^-4^ amino acid changes per site for panel C. Letter a indicates statistically significant difference from no inhibitor control. Letters b and c represent statistically significant change from ethanol (no inhibitor) and BSO (no inhibitor), respectively. (**D–E**) Huh7 cells were transfected with JFH1 RNA and incubated with (D) 0.5% (v/v) ethanol or 20 µM BSO for 48 hrs, or (E) 0.1% (v/v) ethanol or 20 µM BSO±5 µM dipyridyl or 1 mM GSH ester daily for 2 weeks. Then, the samples were analyzed for 8-OHG by ELISA. Data were expressed as fold increase from the control where the control values were 0.106±0.012 and 0.291±0.054 ng/ml for the 48 hr and 2 week time points, respectively. Letters a and b indicate statistically significant difference from the –HCV control and +HCV control, respectively, and c indicates statistically significant difference from respective no inhibitor controls (control no inhibitor, ethanol no inhibitor, and BSO no inhibitor) (n = 4).

Then, whether agents that decrease ROS/RNS can reduce the accelerated mutation rate of HCV was tested. As HCV infection is associated with iron overload [8], cells were incubated with α,α'-dipyridyl, a cell permeable iron chelator, in the presence and absence of ethanol or BSO. Dipyridyl significantly decreased the level of 8-OHG in both ethanol and BSO groups (Fig. 2A). Similarly, GSH ester reduced 8-OHG content in these groups (Fig. 2A). 8-OHG could also be decreased by other iron chelators and inhibitors of nitric oxide synthase, flavoproteins, and CYP2E1 (Fig. S1A). Most of all, dipyridyl decreased the nt. substitution rates of both ethanol and BSO groups so that there was no longer any significant increase over the control (Fig. 2B). The amino acid substitution rate was also significantly attenuated by dipyridyl in these groups (P<0.05) (Fig. 2C). Likewise, GSH ester decreased both nt. and amino acid substitution rates of ethanol and BSO treatment groups (Fig. 2B and 2C).

HCV increases ROS/RNS in hepatocytes [8], [9]. Thus, we next examined whether HCV directly induced oxidative RNA damage in the absence of ethanol and BSO. Huh7 cells were transfected with JFH1 RNA that produces infectious virus particles and exposed to 0.5% ethanol (v/v) or 20 µM BSO daily for 48 hrs, or to 0.1% ethanol (v/v) or 20 µM BSO daily for 2 weeks. HCV significantly increased 8-OHG at both time points, and this was exacerbated by ethanol and BSO (Fig. 2D and 2E). Again, dipyridyl and GSH ester could reduce the ethanol-, BSO-, and HCV-induced elevations in 8-OHG (Fig. 2E). HCV core gene also induced a small but statistically significant increase in 8-OHG (Fig. S1B).

### Ethanol and BSO increase the propensity for antiviral resistance

**Figure 3:**
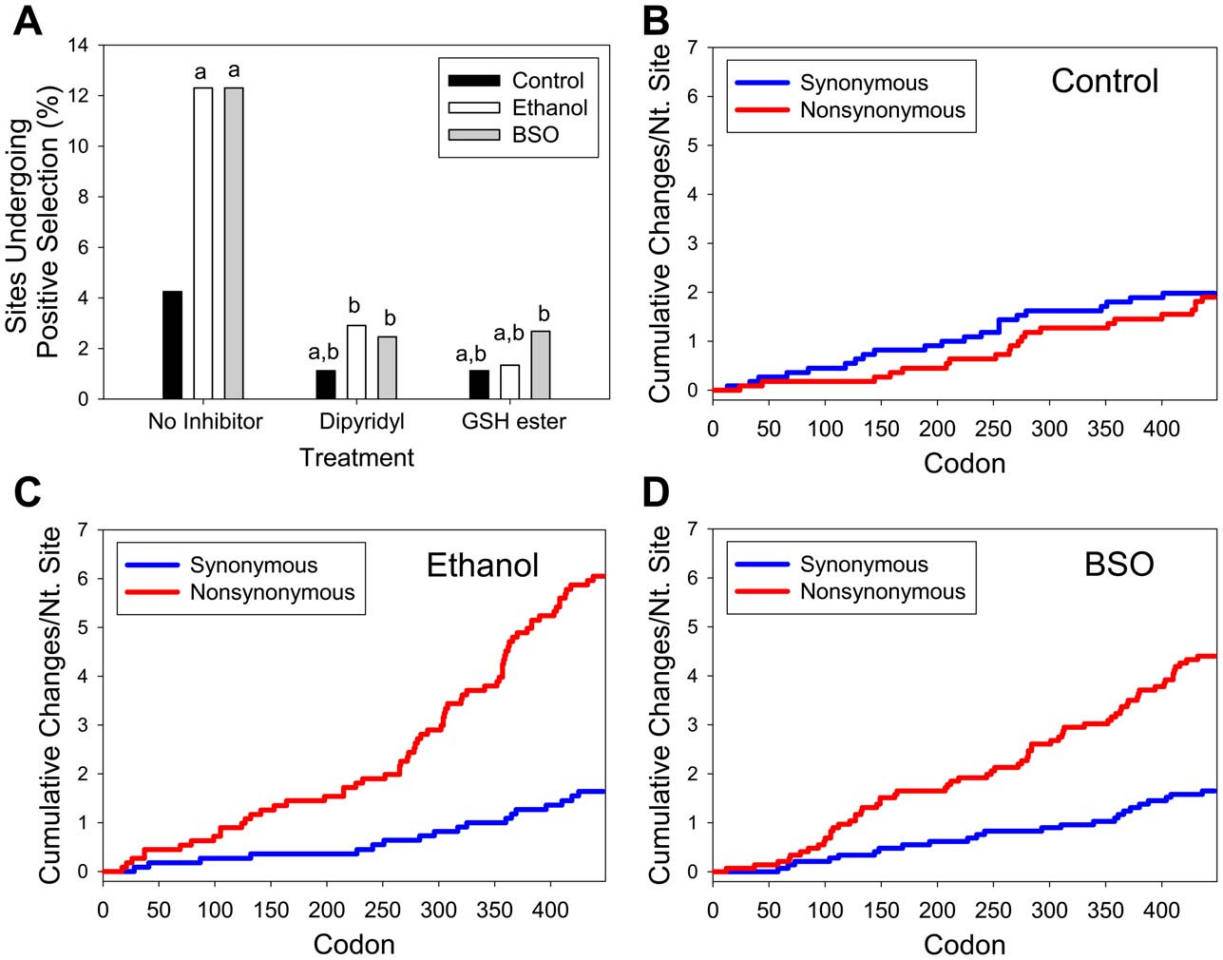
Synonymous and nonsynonymous amino acid substitutions and positive selection in the NS5A coding region. (**A**) Percentage of sites undergoing positive selection (dn/ds ratio>1) in control, ethanol, and BSO groups. Letter a indicates statistically significant difference from no inhibitor control; b indicates statistically significant difference from respective no inhibitor controls (control no inhibitor, ethanol no inhibitor, and BSO no inhibitor). (**B – D**) Cumulative synonymous and non-synonymous mutations in control (B), ethanol (C), and BSO (D) groups were plotted against codons via SNAP. Numbers represent NS5A codon number.

Previously, we and others demonstrated that ethanol, at the concentrations used in the present study, potentiates HCV replication while H_2_O_2_, BSO, and lipid peroxidation products tend to decrease HCV replication [13], [16], [20], [21], [22], [23], [24]. These findings appeared to contradict the mutagenic effects that ethanol can have on HCV RNA under the same conditions (Table 1). Therefore, we next computed the dn/ds ratio to determine whether sequences were undergoing purifying selection, positive selection, or remained neutral during treatments. The dn/ds ratio of our control group was 0.31, and it increased to 1.05 and 0.72 in the ethanol and BSO groups, respectively (Table 1). Percentage of sites showing positive selection also increased from 4.25% in control group to 12.3% in ethanol and BSO groups (Fig. 3A). Average comparisons of 1,212 genotype 1b sequences in the HCV database revealed that 19.5% of the NS5A-coding region experiences positive selection while about 79% undergoes purifying selection. Dipyridyl and GSH ester could each decrease the percentage of sites undergoing positive selection (Fig. 3A). In addition, dipyridyl reduced the dn/ds ratio of the ethanol group from 1.05 to 0.46 and that of the BSO group from 0.72 to 0.46. The dn/ds ratio of the ethanol group dropped from 1.05 to 0.43, BSO group from 0.72 to 0.21, and control group from 0.31 to 0.24, with GSH ester.

Cumulative synonymous and non-synonymous amino acid mutations were also plotted against codons to determine whether mutations clustered at any specific loci (Fig. 3B–3D); vertical steps represent either synonymous or nonsynonymous mutations occurring at that site. As shown in Fig. 3C and 3D, mutations clustered in the C-terminal half of NS5A in ethanol and BSO groups. Sites undergoing positive selection in our study as well as the genotype 1b HCV sequences in the database are mapped in Fig. S2, which shows a greater similarity between the database and our ethanol and BSO treatment groups than between the database and our control group. More specifically, 36.4% of sites undergoing positive selection (dn/ds ratio>1) in our treatment groups were also found to be under positive selection in the database versus 21.1% in the control group. In addition, 36.8% of the sites undergoing positive selection in the HCV database matched sites that underwent positive selection in the ethanol and BSO treatment groups, compared to only 4.6% for the control group. The majority of mutations (97.4%) in our study occurred at sites undergoing positive selection. Synonymous mutations did not increase in the treatment groups (Fig. 3C – D).

**Figure 4:**
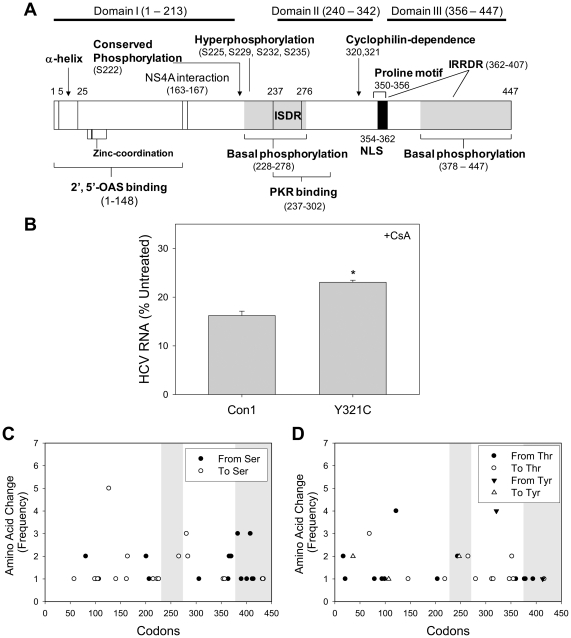
Amino acid substitutions in the NS5A coding region. (**A**) Structural and functional domains of HCV NS5A [17]. Numbers represent NS5A condon number. (**B**) Con1(wt) and Con1(Y321C) replicon cells were treated with 1 mM cyclosporine (CsA) for 48 hrs. Total RNA was extracted and HCV RNA was determined. * Indicates a statistically significant difference. (**C-D**) Location of amino acid substitutions involving serines (C), threonines (D), and tyrosines (D). Putative basal phosphorylation regions are shaded in (A, C, D).

Interestingly, some of the amino acid substitutions found in our study were previously associated with antiviral resistance (Table 2). For example, F37L, which enhances the binding of NS5A to 2',5'-oligoadenylate synthetase, occurred in our study with a dn/ds ratio >1 (Fig. 4A). E442G, V279A, F284S, V445A, E442G, V410A, I302V, and S414P, which confer or otherwise were strongly associated with increased resistance to interferon, ribavirin, cyclosporine, and/or Debio 025 could also be identified (Table 2). In addition, Y321N increases resistance to cyclosporine, and all of the nonsynonymous mutations removing tyrosine in our study occurred at this position (Y321C), and Y321C increased the replicative fitness of HCV in the presence of cyclosporine A (Fig. 4B). Ethanol and reactive species also generated mutations in the CD8+ T cell-specific epitopes previously associated with treatment response (Table 2). Other nonsynonymous amino acid substitutions were found in the binding regions for viral and host proteins including double-stranded RNA-activated interferon induced protein kinase and the putative interferon sensitivity determining regions (ISDR). Four sites within the ISDR which, in combinations, enhanced HCV RNA replication in the study by Kohashi *et al.* had a dn/ds ratio >1 in our study, but no single sequence had more than two amino acid substitutions within ISDR [25]. Amino acid substitutions also occurred in the interferon/ribavirin resistance-determining region (IRRDR) although no single sequence had more than 3 amino acid substitutions within IRRDR [26].

**Table 2:** Functional changes associated with mutations found in this study^a^.

Mutation in Literature	Function^b^	Occurrences in Study^c^	dN/dS>1 at codon^d^	Ref^e^
F37L	Inhibition of 2’,5’-oligoadenylate synthetase	F37L (4)	Yes	[67]
Y321N	Resistance to cyclosporine; conformational change in a major cyclophilin A binding region	Y321C (4)	Yes	[68], [69]
V279A	Resistance to cyclosporine	V279A (2)	No	[70], [71]
F284S	Resistance to cyclosporine	F284S (2), F284L (1)	Yes	[70], [71]
D320E	Resistance to cyclosporine	D320H (1)	No	[69], [70]
V445A	Resistance to Debio 025	V445A (1)	Yes	[70]
E442G	Resistance to IFN	E422G (1), E422K (2), E422V (1)	Yes	[72]
V410A	Resistance to IFN	V410A (2)	Yes	[72]
I302V	Resistance to IFN	I302V (1)	Yes	[72]
S414P	Resistance to IFN	S414P (1)	No	[72]
W433R	Resistance to IFN	W433S (1), W433C (1)	Yes	[72]
S390R	Resistance to IFN	S390N (1)	No	[72]
K277E	Resistance to IFN	K277R (1)	No	[72]
E442G	Resistance to ribavirin	E442G (1)	Yes	[40]
G404S	Resistance to ribavirin	G404D (1)	No	[40]
T21A	Occurred during *in vivo* infection of chimpanzees	T21A (2)	Yes	[73]
M133V	Occurred during *in vivo* infection of chimpanzees	M133V (1)	Yes	[73]
R318H	Occurred during *in vivo* infection of chimpanzees	R318H (1)	Yes	[73]
N69T	Occurred during passage in HEK 293/Sip-L cells	N69T (3)	Yes	[74]
V177I	Occurred during passage in HEK 293/Sip-L cells	V177I (1)	No	[74]
N105Y	Occurred during passage in HEK 293/Sip-L cells	N105S (1), N105D (1), N105G (1)	Yes	[74]
A211E	Occurred during passage in HEK 293/Sip-L cells	A211V (1)	No	[74]
A252V/F	Increased HCV replication	A252V (3)	Yes	[25]
T244I	Increased HCV replication	T244A (2)	Yes	[25]
H247Y	Increased HCV replication	H247Y (2)	Yes	[25]
R246Q	Increased HCV replication	R246L (1)	Yes	[25]
K68R	Increased HCV replication	K68R (2)	No	[75]
L226S	Increased HCV replication	L226S (1)	No	[75]
S264P	Increased HCV replication	E264G (1)	No	[75]
M313A	Increased HCV replication	M313V (1)	Yes	[76]
G341A	Increased HCV replication	G341R (1)	No	[76]

a Mutations from literature were aligned to and listed by the codon numbers used in our study.

b Functions attributed to mutations in the literature.

c Amino acid substitutions occurring at the same position in our study with the number of occurrences shown in parenthesis. Mutations that occurred more frequently are listed in top rows.

d Yes  =  positive selection occurring in our study; No  =  no positive selection.

e Literature describing mutations in column a.

We also found other amino acid substitutions previously reported during *in vivo* infection of chimpanzees as well as some that may affect the replication rate of HCV (Table 2). Notably, 50.3% of all nonsynonymous mutations in our study either added or removed serine, threonine, or tyrosine (STY) in the treatment groups, *versus* 9.5% in the control group (P<0.05). Unlike mutations involving threonine which did not show any obvious clustering, the majority of mutations involving serine in domain 3 tended to remove serine, whereas mutations that generated serine were more frequent in domains 1 and 2 (Fig. 4C and 4D). Some of the STY mutations that occurred more frequently and showed positive selection are listed in Table S2. In comparison, STY substitutions constituted 40.1% of all amino acid variations in the genotype 1b NS5A sequences in the database (Fig. S3). S437 (equivalent to S457 of JFH1), hyperphosphorylation sites, and zinc coordination motif were not changed.

## Discussion

RNA viruses are notorious for pronounced sequence variability and high mutation rates that complicate antiviral therapy and development of vaccines. High mutation rate is attributed to the error-prone viral replicase among other variables. In this study, we used HCV, an important hepatotropic human pathogen, plus ethanol, which is often consumed in relatively high quantities and metabolized to generate reactive species and other reactive metabolites in the liver, to examine how these factors affect the genetic variability of an RNA virus. We found that ethanol and BSO can elevate the basal sequence heterogeneity of HCV RNA in hepatocytes within 48 hrs. Agents that decreased the RNA damage could reduce the sequence variability of HCV. Moreover, infectious JFH1 induced RNA damage even in the absence of ethanol and under GSH-sufficient condition, and this was exacerbated by co-incubation with ethanol or BSO. HCV core has been strongly implicated in oxidative stress, and could induce a small increase in 8-OHG in the absence of other viral factors (Fig. S1B). Endogenous sources of ROS/RNS activated by HCV and HCV core are likely to include the mitochondria, hepatocyte NAD(P)H oxidases, inducible nitric oxidase synthase, as well as CYP2E1 [8], [9], [13]. In addition, effects of iron chelator suggest a role of an iron-dependent process, such as Fenton reaction, in the oxidative RNA damage. These data suggest that HCV genome variability would be affected by endogenous reactive species and GSH depletion occurring *in vivo*, and that reactive species’ accelerating the mutation rate is a direct consequence of HCV infection. These factors, therefore, likely contributed to previous estimation of the mutation rate of HCV in patients. In addition, although the overall quasispecies complexity of HCV is expected to be much higher *in vivo* than in our *in vitro* study that used a single HCV clone, and the magnitude of increase in sequence variability caused by alcohol is also likely to be higher *in vivo* (about 4.5% increase with alcohol in reference [7]) than our *in vitro* study that showed maximal increase in nt. substitution rate of ∼0.14% (from about 0.03 to 0.17% with ethanol, Table 1) and amino acid substitution rate of ∼0.47% (from about 0.03 to 0.5% with ethanol, Table 1), the effects of ethanol observed in our study may have contributed to the increased heterogeneity of HCV RNA reported with alcohol in the studies by Sherman *et al.* and Takahashi *et al*; [6], [7]. In fact, positive selection data from the HCV sequence database were closer to our treatment groups than the control group (Fig. S2), which is consistent with an important role of GSH depletion and endogenous and exogenous sources of reactive species in viral evolution *in vivo*. Any differences between our study and the database (Fig. S2) may be attributed to variable exposure to alcohol, lack of complete viral replication cycle in our replicon cell model, diurnal/hormonal/age-dependent fluctuations in GSH, fluctuations in the level of oxidative/nitrosative stress with viral titer and stage of liver disease, and presence of additional selection pressures *in vivo*.

Our data show that prolonged exposure to low level of ethanol (0.1%, equivalent to 17.2 mM blood alcohol concentration, daily for 2 weeks) could induce RNA damage, as short-term exposure to higher concentration of ethanol (0.5% daily for 48 hrs) also did. Interestingly, prolonged exposure to a low level of ethanol produced less damage than acute exposure to a higher level of ethanol, suggesting that cells adapt to or compensate for chronic repeat exposure to low level of ethanol better than acute exposure to high levels of ethanol. The 0.1% ethanol is approximately the legal limit for drinking under influence in many countries including the U.S.; 0.5% lies in the toxic range but is achieved physiologically, particularly in chronic alcohol users; ethanol is also volatile and the amount metabolized by the cell is significantly less than what is added to cell culture [27]. The concentration of acetaldehyde used in the study also lies within physiological range [28]. These concentrations of ethanol and acetaldehyde did not induce significant cytotoxicity during short-term (48 hr) exposure [13], but prolonged exposure to 0.5% ethanol was not tolerated well by the cell and could not be continued for 2 weeks. As 20 µM BSO is needed in these cells to achieve the level of GSH depletion seen in hepatitis C patients, the same concentration was used in both short-term and long-term experiments. It is known that 70 – 80% reduction in GSH is generally well-tolerated by cells, and BSO did not produce apparent cytotoxicity in our study.

HCV, like other RNA viruses, operates near error threshold and increasing its mutation rate beyond this threshold can lead to an error catastrophe [29], [30]. In this study, both ethanol and BSO induced RNA damage but, as mentioned, H_2_O_2_ and BSO were previously found to suppress HCV RNA replication through calcium modulation whereas the same concentrations of ethanol elevated HCV replication in these cells and in patients [13], [20], [21], [23], [31], [32], [33], [34], [35], [36], [37], [38]. GSH favored HCV replication by countering the suppressive effects of oxidants [14]. While these findings are difficult to reconcile and may appear contradictory, ethanol and oxidative stress/BSO have many effects on the cell, and recently we showed that these seemingly opposite effects of acute ROS/BSO vs. ethanol were explained by other metabolites of ethanol (acetaldehyde, acetate, acetylCoA, and NADH) that had strong lipogenic and therefore favorable effects on HCV that replicates in lipid-rich environment [13]. Furthermore, synonymous mutations did not increase while dn/ds ratio increased with ethanol and BSO treatments in our study. This indicates that mutations we detected were largely due to positive selection. Thus, it is possible that disadvantageous mutations were outcompeted by selection of favorable mutations as the virus adapted to new environment [39]. Indeed, adaptive mutations that affect HCV replication could be detected (Table 2) but nonsense mutations were rare in the study, and it may be speculated that such viral RNAs were outcompeted or eliminated by a process like the nonsense-mediated decay. Importantly, it has also been contested that, under certain conditions, mutagens can impose selection pressure for the development of resistance [39], [40]. Finally, while ethanol and BSO may affect the replicative fitness of individual sequence variants (Table 2, Table S2), it should be noted that frequency of such mutations were still low, and how some of these mutations modulate NS5A phosphorylation and/or viral replication remains to be tested systematically [8], [16], [17], [41]. Thus, overall effects of ethanol and oxidative stress/BSO on viral replication appears to be more biochemically driven, such as by elevation of cytosolic calcium and NADH/NAD+ and cholesterol content, and the significance of increased sequence heterogeneity may lie in increasing the repertoire of sequence variants to facilitate viral escape and persistence (e.g., when subjected to subsequent antiviral therapy), rather than altering the replicative fitness of HCV genome population as a whole via mutations.

In addition to overall increases in the mutation rate, specific NS5A variants previously implicated in antiviral resistance to interferon, ribavirin, cyclosporine, and Debio 025 could be demonstrated in the study (Table 2 and Table S2). Although the frequency of specific mutations was low, these mutations demonstrate specific changes to the HCV genome that may predispose HCV to antiviral resistance. In terms of the mechanism, some of these mutations were most likely generated randomly but positive selection clearly occurred at many of these sites (Table 2). The mechanism by which ethanol and BSO induced positive selection of specific variants remains unknown. Nonetheless, the data strongly suggest that ethanol, reactive species, and certain antivirals have some shared effects so that adaptation to one can affect resistance to another. Here, it may be noteworthy that H_2_O_2_ and BSO have been shown to activate the Jak/Stat pathway in a ligand-independent manner [42], [43]. Mechanism is likely to involve oxidative modification of protein tyrosine phosphatases and modulation of Jak [44], [45]. Ethanol and reactive species can also modulate kinases that have been implicated in NS5A phosphorylation as well as antiviral action of cyclosporine A [12], [46], [47], [48], [49], [50], [51]. In addition to positive selection, it should also be noted reactive species can directly modify amino acids, and oxidative stress as well as viral polymerases can induce transitions or transversions depending on the types of reactive species and polymerase involved [52], [53], [54], [55]. Thus, whether the nucleotide substitution trends purely reflect the most common errors produced by HCV replicase plus positive selection/chemical mutagenesis of viral RNA or also other mechanisms, such as direct chemical modification of the viral replicase by reactive species, remains to be tested.

Therefore, in this study, we showed that ethanol and endogenous reactive species can increase the mutation frequency of HCV in hepatoma cells with evidence of positive selection. Positive selection induced by ethanol and GSH depletion may increase the propensity for antiviral resistance. Our data also suggest that agents that decrease oxidative/nitrosative stress may help attenuate the basal mutation rate of HCV as well as positive selection. Clinically, there is increasing evidence that antioxidants or iron depletion can improve the virological response to IFNα-based therapy, and it will be important to study these interactions further using virus-producing systems *in vitro* as well as *in vivo* under both short-term and long-term exposure conditions, with the inclusion of other regions of HCV genome also implicated in the antiviral resistance [56], [57], [58], [59], [60], [61], [62], [63]. Finally, reactive species will modify RNAs as well as DNAs, and oxidative/nitrosative stress is documented in many different virus infections [64]. Thus, it will be interesting to determine whether some of the findings of this study also apply to other viruses and their physicochemical interactions with the environment affecting viral evolution.

## Materials and Methods

### HCV constructs and cells

Huh7 human hepatoma cell clones supporting continuous replication of a subgenomic HCV replicon RNA of genotype 1b (Con1(S1179I); AJ242652) without forming infectious virions and JFH1 strain (AB047639) that produces infectious virus particles in cell culture were used [16], [65]. Huh7 cells were initially described by Nakabayashi *et al.*, and our Huh7 cell clone metabolizes ethanol by CYP2E1 [13], [66]. The *in vitro* transcription, transfection of the viral RNA, and Huh7 cell culture were performed as described [16], [65].

### Sequencing and sequence analysis

Total RNAs were collected using Trizol (Invitrogen). Then, the NS5A region (nt. 4612–6013 of Con1 replicon) was reverse transcribed/amplified with AccuScript High Fidelity RT-PCR System (Agilent; reverse transcription error rate is 1.61×10^-7^, and DNA polymerase error rate is 4.3×10^−9^). Primer sequences were 5’-ATCAATCGATTGTCTAGAGCTGAAGAGGCTTCACCAG-3’ (reverse) and 5’-GCAATCTTGTACAAGCTTCGCAGCGCATGGCGTGAT-3’ (forward). cDNA products were cloned into pUC19 via HindIII and XbaI sites (underlined) and sequenced at the Joint Genome Institute (Livermore, CA) and University of California Berkeley Sequencing (Berkeley, CA). Sequences were aligned against Con1(S1179I) sequence, using BioEdit/MEGA 4.0 Molecular Evolutionary Genetics Analysis software. Cumulative synonymous and nonsynonymous mutations and dn/ds ratio were computed using Synonymous Nonsynonymous Analysis Program (SNAP; www.hcv.lanl.gov). Amino acid substitution trends were analyzed by position using MutationCounter, a mutation counting program developed for this project, and compared with 1,212 genotype 1b NS5A sequences in the Lawrence Livermore HCV sequence database (www.hcv.lanl.gov). Sequences harboring gaps, undetermined nucleotides, or incomplete sequences were removed prior to the analysis. Sequence data from the Joint Genome Institute and University of California Berkeley Sequencing yielded same conclusions.

### Determination of 8-hydroxyguanosine (8-OHG)

RNA samples were analyzed for 8-OHG by ELISA. Briefly, RNA was incubated with 10 U each of DNase I (GE Healthcare), nuclease P1 (SigmaAldrich or US Biological), and alkaline phosphatase (Promega) and analyzed for 8-OHG using RNA Damage Kit (Cell Biolabs, Inc.).

### Site-directed mutagenesis

An HCV replicon bearing Y321C was generated by site-directed mutagenesis using QuikChange II XL Site-Directed Mutagenesis Kit (Agilent), and sequence was confirmed by DNA sequencing. Primer sequences were 5′-GCACGCCCGGATTGCAACCCTCCACTG-3′ (forward) and 5′-CAGTGGAGGGTTGCAATCCGGGCGTGC-3′ (reverse).

### HCV RNA quantitation

HCV RNA was quantified by quantitative reverse transcriptase polymerase chain reaction, as described [13], [16]. Data were normalized by glyceraldehyde 3-phosphate dehydrogenase mRNA content.

### Statistics

Data were analyzed using Student's t test, one-way analysis of variance, or z-test of proportions using SigmaStat 3.1 (Jandel Scientific). A p value ≤0.05 was considered significant.

## Supporting Information

Figure S1 **RNA damage in Con1 replicon cells and with HCV core. (A)** Con1 replicon cells were incubated with 0.5% ethanol or 20 µM BSO daily for 48 hr with and without 0.1 mM 1,1,4,7,7-diethylenetriaminepentaacetic acid (DETAPAC, an iron chelator), 5 µM pyridoxal isonicotinoyl hydrazone (PIH, an iron chelator), 1 mM N^G^-methyl-L-arginine acetate (L-NMA, inhibitor of nitric oxide synthase), 40 µM diphenylene iodonium (DPI, inhibitor of flavoproteins such as NAD(P)H oxidases), or 25 µM diallyl disulfide (DADS, inhibitor of CYP2E1). Then, the samples were analyzed for 8-OHG by ELISA, as described. Data were expressed as fold increase from the control where the control value was 0.076±0.026 ng/ml. Letter a indicates statistically significant difference from no inhibitor control, and b indicates statistically significant difference from respective no inhibitor controls (control no inhibitor, ethanol no inhibitor, and BSO no inhibitor) (P<0.05; n = 4). All biochemicals were obtained from SigmaAldrich except PIH, which was synthesized (58–59). **(B)** Huh7 cells that are transfected with JFH1 core of genotype 2a, Rcp core of genotype 1a, or empty plasmid vector alone were analyzed for 8-OHG by ELISA. * Indicates statistically significant difference from the control.
(TIF)

Figure S2 **Sites undergoing positive selection, by group. (A)** Location of sites undergoing positive selection in control, ethanol, and BSO groups. Grey lines indicate sites undergoing positive selection that match between this study and the database. Structural and functional domain map of NS5A is drawn approximately to scale. **(B)** Location of sites undergoing positive selection in control, ethanol, and BSO groups, with and without dipyridyl or GSH ester, and the HCV database. Grey lines indicate sites undergoing positive selection that match between this study and the database.
(TIF)

Figure S3 **Position of amino acid variations in the genotype 1b NS5A sequences from database involving serines, threonines, and tyrosines.**
(TIF)

Table S1 **Nucleotide substitution trends in control and treatment groups**.
(DOCX)

Table S2 **Frequent mutations involving serine/threonine/tyrosine residues in the NS5A region.**
(DOCX)
