# Supplementary material for: Ethanol and Reactive Species Increase Basal Sequence Heterogeneity of Hepatitis C Virus and Produce Variants with Reduced Susceptibility to Antivirals
Source: PLoS One. 2011 Nov 8;6(11):e27436. doi: 10.1371/journal.pone.0027436 (PMC3210796; doi:10.1371/journal.pone.0027436)
Supplement: Table S1 — Nucleotide substitution trends in control and treatment groups. (DOCX) [file pone.0027436.s004.docx]

**Table S1.** Nucleotide substitution trends in control and treatment groups^a^

| Nt. substitution | Control | Ethanol | BSO | Database ^b^ |
| --- | --- | --- | --- | --- |
| U→C | 18.18 | 29.87 | 29.49 | 23.57 |
| A→G | 18.18 | 25.97 | 24.36 | 19.62 |
| C→U | 27.37 | 18.18 | 8.97 | 17.58 |
| G→A | 15.15 | 12.99 | 16.67 | 22.7 |
| A→C | 6.06 | 1.30 | 1.28 | 1.54 |
| A→U | 3.03 | 2.60 | 2.56 | 1.97 |
| G→C | 0 | 6.49 | 3.85 | 3.95 |
| G→U | 0 | 2.60 | 3.85 | 2.08 |
| U→A | 0 | 0 | 0 | 3.01 |
| U→G | 3.03 | 0 | 0 | 0.46 |
| C→A | 6.06 | 0 | 3.85 | 1.73 |
| C→G | 3.03 | 0 | 5.13 | 1.77 |

^a^  Data represent percentage of each type of nucleotide substitutions in each group. Transition mutations are shown in the first four rows.

^b^ Percentage of each type of nucleotide substitutions in the genotype 1b HCV sequences in the database compared to Con1 sequence used in the study.
