# Supplementary material for: Ethanol and Reactive Species Increase Basal Sequence Heterogeneity of Hepatitis C Virus and Produce Variants with Reduced Susceptibility to Antivirals
Source: PLoS One. 2011 Nov 8;6(11):e27436. doi: 10.1371/journal.pone.0027436 (PMC3210796; doi:10.1371/journal.pone.0027436)
Supplement: Table S2 — Frequent mutations involving serine/threonine/tyrosine residues in the NS5A region. (DOCX) [file pone.0027436.s005.docx]

**Table S2.** Frequent mutations involving serine/threonine/tyrosine residues in the NS5A region

| Mutation | Study count^a^ | Database  count^b^ | Accession Numbers ^c^ | Reference ^d^ | Type of substitutions, database ^e^ |
| --- | --- | --- | --- | --- | --- |
| F127S | 5 | 79 | AB285036, AF207760, AF265148-50, AF265163-65, AM402055-58, AM402060-78, AM402080-82, AM402084-90, AM402092-110, AM402112-116, AM402118-122, AM402125, AM402127-129, AY808022, DQ491974, DQ492015, EF407482, EF407488,EU482883 | [77,78,79,80,81,82,83,84] | F127S (79), F127C (1), F127L (4), F127Y (2) |
| T122A | 4 | 4 | DQ491961, DQ491964, DQ491966, DQ492018 | [84] | T122A (4), T122K (3), T122R (37), T122V (91), T122W (2) |
| Y321C | 4 | 3 | AM401892, AM401896, AM401974 | [77] | Y321C (3), Y321D (3), Y321N (1) |
| N69T | 3 | 16 | AJ507172, AM402271, DQ491995-97, DQ491999, DQ492000-07, EF407495, HM042050 | [77,78,84,85,86] | N69T (16), N69A (1), N69K (1), N69S (3) |
| S383P | 3 | 10 | AF165061, AF207769, AM401885, AM493449, DQ244140, EF407461, EF407470, EF407482,  EU862837, FJ958916 | [77,78,80,81,87,88,89,90] | S383P (10), S383F (2) |
| S401P | 3 | 39 | AB285042, AF165061-62, AF265158, AF483269, AJ000009, AJ507156, AJ507169, AJ507182, AM493489-507, AY008261, AY808015, AY808026, AY808030-31, AY808043, DQ492060, DQ492152, EU155281, EU256062, EU256080 | [79,80,82,83,84,86,88,89,91,92,93] | S401P (39), S401A (2), S401F (6), S401L (39), S401T (1) |
| S408P | 3 | 3 | AM401886, AM402364, AM402374 | [77] | S408P (3), S408F (1), S408T (5), S408Y (1) |
| T379A | 2 | 64 | AB049092, AB285040, AF165045-46, AF207755, AM402055, AM402060, AM402066, AM402070-71, AM402074-75, AM402081, AM402088, AM402090, AM402096, AM402098, AM402101-102, AM402106, AM402108, AM402111-112, AM402115, AM402125, AM402127, AY460204, EF407470, EF407477, EU155218, EU155381, EU862837, EU781832, HM042053 | [77,78,79,80,81,85,88,94,95] | T379A (64), T379S (7) |

^a^ Frequency (count) of substitutions occurring in the treatment groups.

^b^ Frequency of substitutions in the genotype 1b HCV sequences in the database compared to Con1 sequence used in the study.

Positive selection occurred at F127, T122, T379, and S401 in the database.

^c^ Accession numbers for HCV sequences in column ^b^.

^d^ References for HCV sequences in columns ^b^ and ^c^.

77. Chevaliez S, Brillet R, Lazaro E, Hezode C, Pawlotsky JM (2007) Analysis of ribavirin mutagenicity in human hepatitis C virus infection. J Virol 81: 7732-7741.

78. Donlin MJ, Cannon NA, Yao E, Li J, Wahed A, et al. (2007) Pretreatment sequence diversity differences in the full-length hepatitis C virus open reading frame correlate with early response to therapy. J Virol 81: 8211-8224.

79. El-Shamy A, Sasayama M, Nagano-Fujii M, Sasase N, Imoto S, et al. (2007) Prediction of efficient virological response to pegylated interferon/ribavirin combination therapy by NS5A sequences of hepatitis C virus and anti-NS5A antibodies in pre-treatment sera. Microbiol Immunol 51: 471-482.

80. Kuntzen T, Timm J, Berical A, Lennon N, Berlin AM, et al. (2008) Naturally occurring dominant resistance mutations to hepatitis C virus protease and polymerase inhibitors in treatment-naive patients. Hepatology 48: 1769-1778.

81. Nagayama K, Kurosaki M, Enomoto N, Miyasaka Y, Marumo F, et al. (2000) Characteristics of hepatitis C viral genome associated with disease progression. Hepatology 31: 745-750.

82. Nousbaum J, Polyak SJ, Ray SC, Sullivan DG, Larson AM, et al. (2000) Prospective characterization of full-length hepatitis C virus NS5A quasispecies during induction and combination antiviral therapy. J Virol 74: 9028-9038.

83. Veillon P, Payan C, Gaudy C, Goudeau A, Lunel F (2004) [Mutation analysis of ISDR and V3 domains of hepatitis C virus NS5A region before interferon therapy with or without ribavirin]. Pathol Biol (Paris) 52: 505-510.

84. Veillon P, Payan C, Le Guillou-Guillemette H, Gaudy C, Lunel F (2007) Quasispecies evolution in NS5A region of hepatitis C virus genotype 1b during interferon or combined interferon-ribavirin therapy. World J Gastroenterol 13: 1195-1203.

85. Kumthip K, Pantip C, Chusri P, Thongsawat S, O'Brien A, et al. (2010) Correlation between mutations in the core and NS5A genes of hepatitis C virus genotypes 1a, 1b, 3a, 3b, 6f and the response to pegylated interferon and ribavirin combination therapy. J Viral Hepat 18: e117-124.

86. Sarrazin C, Herrmann E, Bruch K, Zeuzem S (2002) Hepatitis C virus nonstructural 5A protein and interferon resistance: a new model for testing the reliability of mutational analyses. J Virol 76: 11079-11090.

87. Jain MK, Yuan HJ, Adams-Huet B, Reeck A, Shelton J, et al. (2009) Pegylated interferon and ribavirin promote early evolution of nonstructural 5A protein in individuals with hepatitis C who demonstrate a response to treatment. J Infect Dis 200: 866-876.

88. Nagayama K, Kurosaki M, Enomoto N, Maekawa SY, Miyasaka Y, et al. (1999) Time-related changes in full-length hepatitis C virus sequences and hepatitis activity. Virology 263: 244-253.

89. Piodi A, Chouteau P, Lerat H, Hezode C, Pawlotsky JM (2008) Morphological changes in intracellular lipid droplets induced by different hepatitis C virus genotype core sequences and relationship with steatosis. Hepatology 48: 16-27.

90. Yagi S, Mori K, Tanaka E, Matsumoto A, Sunaga F, et al. (2005) Identification of novel HCV subgenome replicating persistently in chronic active hepatitis C patients. J Med Virol 77: 399-413.

91. Polyak SJ, Khabar KS, Paschal DM, Ezelle HJ, Duverlie G, et al. (2001) Hepatitis C virus nonstructural 5A protein induces interleukin-8, leading to partial inhibition of the interferon-induced antiviral response. J Virol 75: 6095-6106.

92. Trowbridge R, Gowans EJ (1998) Molecular cloning of an Australian isolate of hepatitis C virus. Arch Virol 143: 501-511.

93. Yildiz E, Oztan A, Sar F, Pinarbasi E, Cetin-Atalay R, et al. (2002) Molecular characterization of a full genome Turkish hepatitis C virus 1b isolate (HCV-TR1): a predominant viral form in Turkey. Virus Genes 25: 169-177.

94. Mao HX, Hu YW, Wu Y, Lan SY, Yuan ZH (2004) [Construction of full-length complementary DNA of hepatitis C virus genome from an HCV infected patient]. Zhonghua Shi Yan He Lin Chuang Bing Du Xue Za Zhi 18: 122-126.

95. Takahashi K, Iwata K, Matsumoto M, Matsumoto H, Nakao K, et al. (2001) Hepatitis C virus (HCV) genotype 1b sequences from fifteen patients with hepatocellular carcinoma: the 'progression score' revisited. Hepatol Res 20: 161-171.

^e^ Type of amino acid substitutions occurring at that position in the genotype 1b HCV database and frequencies (count), shown in parenthesis.
